# Inherited prion disease caused by a novel frameshift mutation of *PRNP* resulting in protein truncation at codon 157

**DOI:** 10.1177/13872877251351182

**Published:** 2025-07-04

**Authors:** Leah Holm-Mercer, Tze How Mok, Danielle Sequeira, Thomas Coysh, Peter Rudge, Hawraman Ramadan, Lee Darwent, Tracy Campbell, Thomas Murphy, Colin Smith, Diane Ritchie, Sebastian Brandner, Zane Jaunmuktane, John Collinge, Simon Mead

**Affiliations:** 1 MRC Prion Unit at UCL, UCL Institute of Prion Diseases, University College London, London, UK; 2 NHS National Prion Clinic, National Hospital for Neurology and Neurosurgery, University College London Hospitals NHS Foundation Trust, London, UK; 3 1906 Bradford Teaching Hospitals NHS Foundation Trust, Bradford Royal Infirmary, Bradford, UK; 4 Academic Department of Neuropathology, Centre for Clinical Brain Sciences, University of Edinburgh, Edinburgh, UK; 5 Department of Neurodegenerative Disease, 61554 UCL Queen Square Institute of Neurology, University College London, London, UK; 6 Division of Neuropathology, National Hospital for Neurology and Neurosurgery, London, UK; 7 Department of Clinical and Movement Neurosciences, 61554 UCL Queen Square Institute of Neurology, University College London, London, UK

**Keywords:** Alzheimer's disease, cerebral amyloid angiopathy, frameshift mutation, neurofibrillary tangles, prion diseases

## Abstract

**Background:**

PrP systemic amyloidosis is increasingly recognized as a novel inherited prion disease (IPD) syndrome caused by *PRNP* C-terminal truncating mutations. As well as systemic manifestations they cause gradually progressive cognitive impairment with neurofibrillary tangle pathology which can be mistaken for Alzheimer's disease (AD).

**Objective:**

We describe the clinical, biomarker and neuropathological features of a novel frameshift mutation of *PRNP* resulting in protein truncation at codon 157.

**Methods:**

The clinical phenotype and biomarker findings, including plasma biomarkers measured using Single Molecule Array (SiMOA) technology are reported for affected living individuals, with neuropathological examination available for the index case.

**Results:**

The Y157X *PRNP* mutation has resulted in a phenotype of gradually progressive cognitive decline, peripheral sensory and autonomic polyneuropathy, and gastrointestinal symptoms, with one case presenting with recurrent episodes of nausea, vomiting and electrolyte derangement requiring intensive care unit admission. Plasma biomarkers revealed an AD-like pattern with raised neurofilament light chain (NfL), glial fibrillary acidic protein (GFAP) and phospho-tau 181 (P-tau 181) in affected individuals. On neuropathological examination there was PrP-cerebral amyloid angiopathy (CAA) and neurofibrillary tau pathology.

**Conclusions:**

We present the clinical, biomarker and pathological findings on investigation of this family and provide further evidence for the association of truncation mutations with PrP systemic amyloidosis.

## Introduction

Prion diseases are a group of fatal transmissible neurodegenerative diseases in which the infectious agent is composed of assemblies of abnormally folded host-encoded prion protein (PrP). Misfolded PrP is heterogeneous in its aggregation state and can consist of amyloid plaques, more diffuse deposits, or soluble species. Inherited prion diseases (IPD) account for 10–15% of the incidence of prion diseases; these are all caused by coding mutations in the prion protein gene (*PRNP*)^1^ and are inherited in an autosomal dominant fashion.

The clinical phenotype of IPD is highly variable both within and between families. Several canonical syndromes are readily recognized, including familial Creutzfeldt-Jakob disease (fCJD), fatal familial insomnia (FFI), Gerstmann-Straussler-Scheinker disease (GSS) and more recently PrP systemic amyloidosis.^2^ The latter phenotype, characterized by hereditary sensory and autonomic neuropathy caused by C-terminal domain truncation mutations of PrP, is associated with systemic amyloidosis, and has now been described in several families.^2–14^ The presence of PrP amyloid was demonstrated for Y163X,^2^ and given the phenotypic and pathological similarities between Y163X and the other truncation mutations, the presence of PrP amyloid in PrP systemic amyloidosis phenotypes is inferred on this basis. Infectivity has not been demonstrated in PrP systemic amyloidosis.^2^

Here we describe a family presenting with chronic diarrhea, autonomic and sensory neuropathy and progressive cognitive impairment with neuropathology showing PrP amyloid plaques, cerebral amyloid angiopathy (CAA) and cortical tau disease in the form of neurofibrillary tangles. Genetic analysis revealed a novel frameshift mutation of *PRNP* resulting in truncation at codon 157 (c.[470dup];[=] p.[(Tyr157*)];[(=)]). This mutation strengthens the evidence for an association between C-terminal truncation mutations of *PRNP* and the PrP systemic amyloidosis phenotype and we provide results of plasma biomarkers.

## Methods

### Patients

Cases IIIa and IIIc underwent thorough clinical investigation and are enrolled to the National Prion Monitoring Cohort (Scotland A Research Ethics Committee (Ref: 05/MRE/0063)). For case IIa details were obtained from archived clinical notes, and interview of family members.

### Biomarkers

Plasma glial fibrillary acidic protein (GFAP), neurofilament light chain (NfL), Tau, and ubiquitin C-terminal hydrolase L1 (UCH-L1), and plasma phosphorylated tau-181(P-tau 181) were measured by Single Molecule Array (SiMOA) technology on the HD-X analyzer, using the Neurology 4-plex B platform, as per manufacturer's instructions. This was part of 2 datasets, one a large cohort of individuals at risk of, or symptomatic of prion disease against normal controls published elsewhere,^15^ and the other a cohort of individuals with symptomatic prion disease against normal controls which is unpublished.

### Neuropathology

Formalin-fixed and paraffin-embedded post mortem brain tissue blocks from the cingulate gyri with corpus callosum, anterior hippocampus, basal ganglia, pons, medulla and cerebellum, were re-examined by re-staining for hematoxylin and eosin, and immunostained for abnormal prion protein (D-Gen Ltd, London, UK, ICSM35,1:1000 and Cayman Chemical, UK 12F10, 1:200), amyloid-β (DAKO; M0872; 6F3D; 1:50), hyperphosphorylated tau (Invitrogen; MN1020; AT8; 1:1200), non-phosphorylated TDP43 (Abnova, 2E2-D3, 1:500) and p62 (BD Transduction; 610833; 3/P62LCK Ligand; 1:100). Immunostaining was performed on a ROCHE Ventana Discovery automated staining platform following the manufacturer's guidelines, using biotinylated secondary antibodies and a horseradish peroxidase-conjugated streptavidin complex and diaminobenzidine as a chromogen. All immunostainings were performed with appropriate controls. All slides were digitized on a digital slide scanner at x40 magnification (NanoZoomer S360, Hamamatsu) and for pathology figure preparation representative images were taken on NZConnect (Hamamatsu) slide viewing platform.

### Genetic analysis

Blood for DNA extraction from Case IIIa and IIIc was obtained and genomic DNA extracted following appropriate genetic counselling. Sanger sequencing of *PRNP* was performed as described previously.^16^

## Results

### Clinical history

*Case IIa*. Case IIa was a white British man who died aged 50 from a deep vein thrombosis with pulmonary embolism after an illness spanning 5 years. Initial assessment by a neurologist revealed cognitive impairment and gait ataxia, and his neuropsychometric profile was consistent with an Alzheimer's disease pattern. He later became symptomatic of postural hypotension, resulting in frequent syncopal attacks. Family members recalled in retrospect he did have bowel disturbance and distal lower limb sensory loss, but did not seek any medical explanation for these symptoms.

*Case IIIa*. Case IIIa is a right-handed white British man who presented aged 52 with a two-year history of gradual cognitive decline, beginning first with episodic confusion, followed by failure in navigating home from work, inability to recognize family members, and word finding difficulty. Sensory loss in his feet and urinary incontinence then surfaced, but without reports of recurrent urinary tract infections or symptomatic renal calculi. He went on to develop an unusual circumferential (5 cm diameter) ulcer with a necrotic base and erythematous edges over his right greater trochanter area. Appearances were not in keeping with a pressure sore, and did not cause pain. This was complicated by osteomyelitis requiring admission for intravenous antibiotics.

He has a history of glaucoma, and chronic diarrhea diagnosed as irritable bowel syndrome (IBS), for which he took mebeverine 135 mg twice a day.

On examination he had postural hypotension (>20 mmHg systolic blood pressure) recorded on several occasions. Neurological examination revealed subtle left sided cerebellar signs, absent ankle jerks, impaired distal lower limb pinprick perception up to the dorsum of his feet and impaired temperature perception up to the knee on the left, and shin on the right. Light touch vibration and joint position perceptions were normal. A bladder scan showed a post void residual volume of 500 mL.

Bedside cognitive examination revealed impaired naming of line drawings and reduced verbal fluency, as well as mildly impaired verbal recognition, acalculia, and a reduced forward digit span. Recognition of fragmented letters was intact. He displayed both ideational and ideomotor apraxia.

Nerve conduction studies (NCS) revealed a length-dependent sensory axonal neuropathy restricted to his lower limbs. There were unobtainable sympathetic skin responses in the right foot and unobtainable sensory responses on left superficial peroneal nerve stimulation, with small sensory nerve action potentials on right superficial peroneal nerve stimulation (3.1 µV, normal range ≥ 4 µV) and bilaterally on sural nerve stimulation (right 3.6 µV, left 2.6 µV, normal range ≥ 7µV) with normal conduction velocities (right superficial peroneal nerve 42.0 m/s, right sural nerve 41.4 m/s, left sural nerve 39.9 m/s, normal range ≥ 40 m/s). Sensory responses were of normal amplitude in the upper limbs (right radialis 29.6 µV, normal range ≥ 15 µV, conduction velocity 54.1 m/s, normal range ≥ 50 m/s) with normal motor studies (recorded from peroneal, tibial and ulnar motor nerves). MRI brain showed normal cerebral volume for age, and no diffusion-weighted imaging changes were seen. Cerebrospinal fluid (CSF) examination and electroencephalogram (EEG) were not done.

Supportive and symptom management remain the cornerstones of his clinical care plan including podiatry and continence care, the latter requiring long term urinary catheterization for his neurogenic bladder. Prolonged release galantamine 16 mg once a day appeared to result in anecdotal improvement according to his mother. Postural hypotension has been largely asymptomatic to date and managed conservatively with advice about postural changes.

*Case IIIc*. Case IIIc is a right-handed white British female who presented aged 45 following predictive *PRNP* testing which revealed the presence of *PRNP* Y157X. She had a long history of bowel disturbance (episodic loose stool associated with crampy abdominal pain and fecal urgency) beginning aged 19, initially diagnosed as IBS, for which she took mebeverine for symptomatic relief. She had a history of episodes of severe nausea and vomiting associated with electrolyte disturbance and weight loss (twice aged 44 and 45 concurrent with testing positive for covid-19, and once when suffering from hyperemesis gravidarum for 7 months of her pregnancy). Since childhood she had been prone to fainting, with triggers including standing up too quickly, anxiety and stress. She began to report increased urinary frequency but no urinary urgency or urinary tract infections and subsequently went on to develop progressive short-term memory and word-finding difficulties, and increasingly frequent episodes (approximately 8 episodes in 14 months) of nausea, vomiting, electrolyte disturbance (hypokalemia and hyponatremia), symptomatic postural hypotension and delirium requiring hospital and sometimes intensive care admissions. Between these episodes she reported no gastrointestinal symptoms or symptomatic postural hypotension.

On examination her BMI was 18.8 kg/m^2^. She had a postural drop from 158/112 mmHg sitting to 138/111 mmHg standing. On neurological examination she had hypersensitivity in her feet with mild impairments in temperature sensation in the lower limb up to her mid-shins and altered sensation to pin prick on the dorsum of her feet, with no other significant findings. Bedside cognitive examination revealed impaired naming of line drawings and reduced verbal recognition memory and mild ideational apraxia. Neuropsychometry (during delirium) showed severe cognitive impairment predominantly affecting temporal and frontal regions.

During a hospital admission aged 45 with nausea and vomiting, blood biochemistry changes included hypokalemia (2.6 mmol/L) and hyponatremia (124 mmol/L) with endocrine tests suggestive of an element of syndrome of inappropriate ADH (SIADH) worsened by vomiting. CSF examination revealed raised protein (0.8 g/L), CSF/serum albumin ratio (13.5), neurofilament-light (NfL) (1576 pg/mL, normal range 0–967 pg/mL), total tau (>2000 pg/mL, normal 146–595 pg/mL) and phosphorylated tau-181 (>400 pg/mL, normal 0–58 pg/mL) with normal glucose, white cell count, beta-amyloid 1–42 and 1–40 and negative oligoclonal bands. Autonomic plasma catecholamine testing showed slightly low basal plasma catecholamine levels with no rise on tilting consistent with post ganglionic involvement, with autonomic function testing revealing orthostatic hypotension and reduced heart rate variability in response to breathing. MRI brain showed more white matter hyperintensities than expected for age, but no evidence of micro-hemorrhages on SWI sequences suggestive of CAA. On NCS there was a length-dependent sensory and autonomic small fiber polyneuropathy. Warm and cold detection and sympathetic skin responses were absent in the feet with elevated warm detection (41.7°C, normal range 32°C-40°C) and borderline cold detection (29.4°C, normal range 28°C–31°C) in her hands; the study was otherwise normal (sensory responses in the sural nerve 18 µV (normal range ≥ 7 µV) and superficial peroneal 22 µV (normal range ≥ 4 µV) with conduction velocities of 56 m/s and 58 m/s respectively (normal range ≥ 40 m/s), normal motor studies recorded from tibial, ulnar and common peroneal nerves). EEG showed widespread underlying/intermixed theta and slow activity with more prominent slow wave bursts. An endoscopy was normal and light microscopy of stomach and small bowel biopsies revealed no evidence of amyloid deposition.

In this case symptomatic management of acute episodes of nausea, vomiting and electrolyte disturbance included fluid resuscitation and electrolyte replacement with regular ondansetron and cyclizine. Regular potassium supplementation, cyclizine and high dose omeprazole were started to prevent re-occurrence of episodes. Symptoms due to peripheral sensory and autonomic neuropathy are managed conservatively.

### Family history

**Figure 1.**
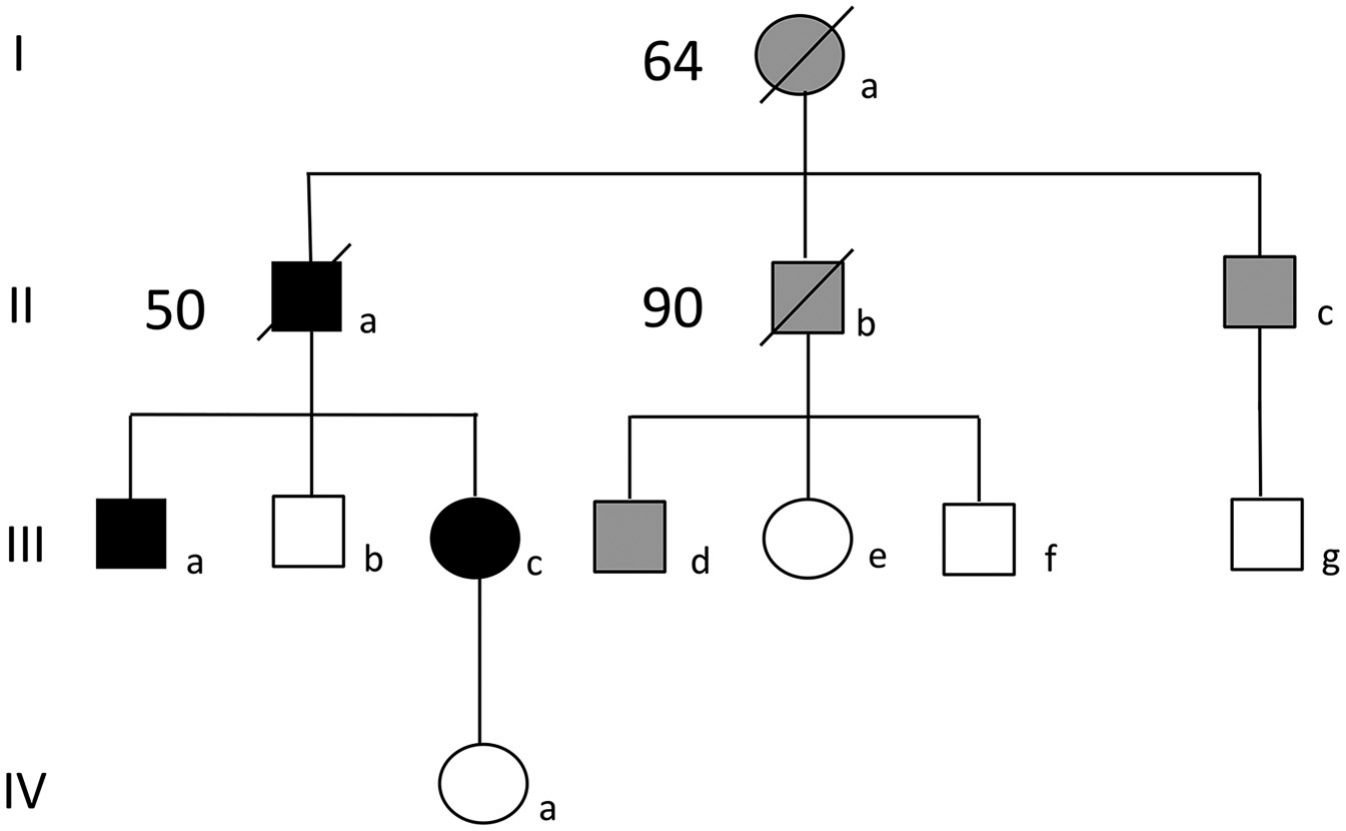
Y157X family pedigree. Squares indicate male, circles indicate female. The number beside symbols with a slash is age at death. Black symbols show affected family members. Gray symbols show family members with a diagnosis of a neurodegenerative disease.

Case Ia had a diagnosis of dementia and died aged 64. Cases IIb and IIc have both developed dementia in their late 80 s. Case IIId received a diagnosis of Parkinson's disease in his 30 s (Figure 1).

### Genetic analysis

Sequencing of *PRNP* in DNA samples obtained from Case IIIa and IIIc revealed a novel single base frameshift mutation of *PRNP* resulting in a stop codon and protein truncation at codon 157 (c.[470dup];[ = ] p.[(Tyr157*)];[(=)]). The polymorphism at codon 129 genotype was methionine valine heterozygous in both cases with the mutation in cis with valine at codon 129.

### Blood biomarkers

Blood biomarker results are displayed with normal range as study control means +/- two standard deviations (data normalized to age 60 when there was a significant age effect). For patient IIIa (at age 54) plasma NfL and ptau-181 were elevated at 68.08 pg/mL (normal range 0.07–24.51 pg/mL) and 130.62 pg/mL (normal range 2.96–41.82 pg/mL) respectively. This was also the case for patient IIIc (at age 45) with NfL at 30.27 pg/mL (normal range 0.07–24.51 pg/mL) and P-tau 181 at 78.74 pg/mL (normal range 2.96–41.82 pg/mL). GFAP, Tau and UCH-L1 were also measured in Patient IIIa on 2 occasions 6 months apart (aged 52), with only GFAP outside the normal range at 16.44 pg/mL and 15.64 pg/mL (normal range 4.31–9.61 pg/mL).

### Neuropathology

**Figure 2.**
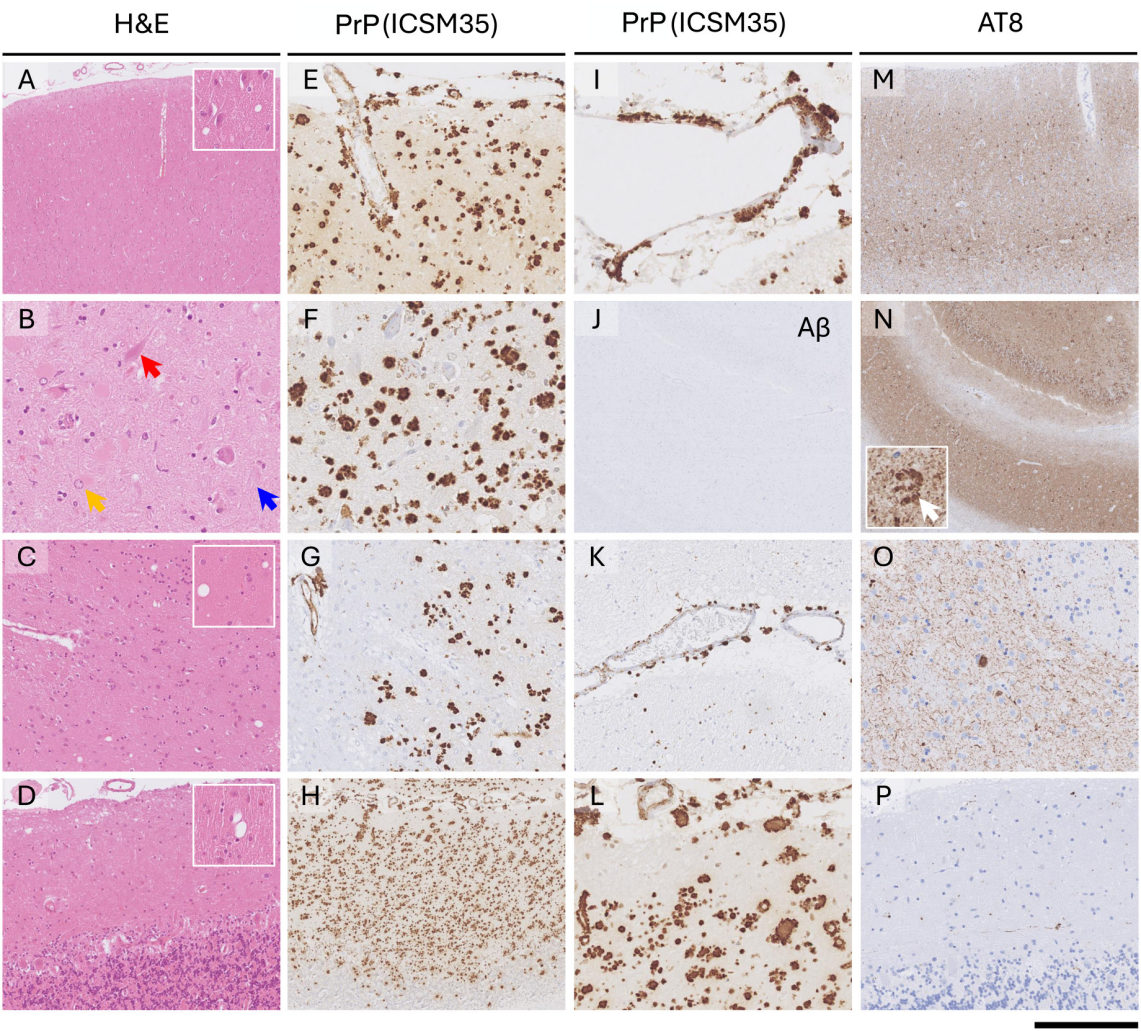
Hematoxylin and eosin (H&E) stained sections **(**A-D) from the (A) cingulate cortex shows no spongiform degeneration, whilst (B) in the CA1 hippocampal region, there is a very severe neuronal depletion with readily visible neurofibrillary tangles (red arrow) and ghost tangles (blue arrow), accompanied by prominent reactive astrogliosis (yellow arrow). (C) in the caudate nucleus, neuronal depletion is minimal (highlighted in the inset). (D) In the cerebellar cortex, there is mild gliosis in the molecular layer, and patchy Purkinje cell depletion. Immunostaining for abnormal prion protein with ICSM35 antibody (E-H, I, K, L) shows frequent micro-plaques across all gray matter regions, with moderately frequent prion protein cerebral and cerebellar amyloid angiopathy seen in the leptomeninges and gray matter. (J) Immunostaining for amyloid-beta is entirely negative in the parenchyma and blood vessel walls. (M-P) There is a dense hyperphosphorylated tau pathology, demonstrated with AT8 antibody, with a dense meshwork of neuropil threads, frequent neurofibrillary tangles and neuritic plaques seen in the cingulate cortex (M), across the hippocampus (N, the white arrow in the inset highlights a neuritic plaque from CA4 hippocampal region, containing central core of PrP abnormal deposit), and with looser meshwork of neuropil threads and occasional pre-tangles seen in the caudate nucleus (O) and as rare long neurites in the cerebellar molecular layer (P). There was neuronal cytoplasmic TDP43 pathology colocalizing with neurofibrillary tau tangles, restricted to the hippocampus (not shown). No severe cortical atrophy or spongiform degeneration was present in any of the gray matter regions. Scale bar: 600 µm in J and N; 400 µm in A, D, H, M, 200 µm in C, E, G, 100 µm in B, F, I, K, L, O, P.

Neuropathology from Case IIa (Figure 2) showed misfolded prion protein pathology in the form of numerous densely packed micro-plaques across all examined gray matter regions. There is also up to moderately frequent misfolded prion protein cerebral amyloid angiopathy seen in cerebral and cerebellar leptomeninges and across the gray matter affecting all caliber arterioles, venules and rare capillaries. Misfolded prion protein pathology was accompanied by widespread neurofibrillary tangle and neuropil thread tau pathology, seen across all hippocampal regions, cingulate cortex, deep gray nuclei regions and also as rare long neurites in the cerebellar molecular layer. Restricted to the hippocampus, there was also TDP43 neuronal cytoplasmic pathology, colocalizing with hyperphosphorylated tau bearing neurons. P62 immunoreactivity was restricted to tau pathology. There was no amyloid-beta parenchymal or vascular pathology. There was no alpha-synuclein pathology based on absent p62 immunoreactivity, although it should be noted that this was not directly assessed with alpha-synuclein specific antibodies.

## Discussion

We report a family with an autosomal dominant inherited prion disease caused by a novel PrP truncating mutation Y157X, characterized by features of a systemic amyloidosis (peripheral sensory and autonomic neuropathy, gastrointestinal symptoms), slowly progressive cognitive decline and neuropathology demonstrating PrP-CAA and hyperphosphorylated-tau pathology. Plasma P-tau 181 was markedly raised in the two tested cases. The clinical features and neuropathology here bear striking similarities to that observed in other truncation mutations leading to PrP systemic amyloidosis. Gut, bladder and vascular dysautonomia, and length-dependent neuropathy appear to be commonly shared clinical features, reflecting abundant PrP amyloid deposition in the central nervous system, vascular system and in other affected peripheral tissues on autopsy.^2^ The neuropathy seen in this pedigree affects mostly autonomic pathways in contrast to the peripheral neuropathy reported in sporadic CJD cases,^17^ suggesting different pathogenetic mechanisms in PrP systemic amyloidosis.

Elevated plasma P-tau 181 levels in IIIa and IIIC and CSF in IIIc, in the absence of beta amyloid pathology in CSF (IIIC) and at neuropathology (IIa), align with findings of elevated CSF and plasma P-tau 181 and P-tau 217 in sporadic CJD.^18^ Given the young age of IIIa and IIIc the elevated P-tau is likely to represent a secondary rather than primary tauopathy, supporting the notion that P-tau elevation may be a marker of extra-neuronal amyloid accumulation and should not just be considered as reflective of AD-amyloid associated changes in tau phosphorylation. This underscores the need for careful interpretation of biomarker findings in accurate AD diagnosis.

Truncating mutations generate a stop codon that signals the end of protein synthesis, with subsequent interruption of mRNA translation and expression of an incomplete or truncated protein. In *PRNP* this results in loss of the glycosylphosphatidylinositol (GPI)-anchor that tethers PrP to the cell membrane. Truncating mutations of the C-terminal domain of PrP have previously been described in patients at codons 145,^3,4^ 160,^5,6,8,19^ 162^9^ 163,^2,10–12^ 169,^12^ 178,^13^ 226^14^ and 227^14^ (Table 1). The clinical phenotypes (symptoms expression and the order in which they emerge) vary between truncation mutations, and even within members of the same family with the same mutation.

**Table 1.** Describes the clinical and neuropathological features of C-terminal *PRNP* truncating mutations which have been described in the literature.

Reference	Mutation	c129	Clinical phenotype	Neuropathology
Kitamoto et al.^3^	Y145X	Not reported	Alzheimer's disease-like dementia from 38 years old. Severe cerebral atrophy on CT.	Many PrP amyloid plaques, no spongiform change. Diffuse neuropil threads of paired helical filaments.
Ghetti et al.^4^	Y145X	MM	Slowly progressive dementia, age of onset 38. Same case as reported in Kitamoto et al.	Abundant neurofibrillary changes, and PrP-CAA. No spongiosis. Same case as reported in Kitamoto et al.
Finckh et al.^5^	Q160X	Proband: MM Brother: MV (mutation associated with M)	Early onset dementia in the proband from age 32 and in the proband's sibling at age 48. Father died aged 60, developed dementia aged 48.	None available
Jayadev et al.^6^	Q160X	Proband: MV Mother: MM	Alzheimer's disease-like dementia, proband's age at onset 38. The proband's mother died aged 67, following an 8-year history of dementia. The mother also had history of weight loss and post-prandial diarrhea 2 years after the onset of cognitive and behavioral changes	PrP amyloid plaques with severe tau neurofibrillary tangles and neuritic plaques. Staining for A-beta and TDP-43 negative.
Guerreiro et al.^19^	Q160X	Proband: VV	The proband had Alzheimer's disease-like dementia from age 38. Background of erectile dysfunction. Temporary diarrhea thought to be related to introduction of Arecept. Mother of the proband had similar early onset cognitive impairment.	None available
Fong et al.^8^	Q160X	Proband: MM Father: MV Paternal cousin: MM	Onset in proband at age 27 - orbitofrontal syndrome, cyclic diarrhea, peripheral neuropathy followed by memory predominant cognitive syndrome. Father asymptomatic (age 56). Paternal uncle had history of autonomic/cognitive illness with neuropathy/diarrhea. 50-year-old paternal cousin had similar symptoms to proband.	None available
Matsuzono et al.^9^	Y162X	Not reported	Diarrhea from age 35, urinary retention and secondary pyelonephritis, orthostatic hypotension, esophageal achalasia. Similar symptoms in mother and aunt.	Lower esophageal biopsy: coarse granular deposits of PrP along basal membrane
Revesz et al.^10^	Y163X - unpublished data	Not reported	Not described	Vascular and parenchymal disease-associated PrP deposition and extensive neuro-fibrillary tangle pathology
Mead et al.^2^	Y163X	Mutation associated with V	Chronic diarrhea, peripheral neuropathy, and autonomic failure. Cognitive decline/seizures when in 40s/50s	Cortical amyloid plaques, CAA and tauopathy. PrP-amyloid seen throughout peripheral organs
Capellari et al.^12^	Y163X/Y169X	Y163X - mutation associated with V Y169X - mutation associated with M	Disease typically presented in the third or fourth decade with progressive autonomic failure and diarrhea. Moreover, one proband (p.Y163X) developed late cognitive decline, whereas some of his relatives presented with isolated cognitive and psychiatric symptoms	None
Themistocleous et al.^11^	Y163X	Not reported	Peripheral neuropathy, autonomic dysfunction, diarrhea	None
Matsuzono et al.^13^	178X	Not reported	Proband presented with urinary retention at age 26 followed by orthostatic hypotension aged 30. One year later developed memory disturbances followed by vomiting and diarrhea. Mother developed dementia, urinary disturbance and orthostatic hypotension at the age of 47 years with thermoanesthesia in her lower limbs, dying one year after symptom onset. The grandfather of the proband developed dementia with urinary disturbance and orthostatic hypotension aged 52 and died age 62.	None
Jansen et al.^14^	Y226X/ Q227X	Y226X – MV (mutation associated with V) Q227X – MV (mutation associated with V)	Y226X – died age 57 after 27-month illness. Dementia with visual and auditory hallucinations Q227X – dementia with slowly progressive hypokinetic rigid syndrome	Y226X – PrP-CAA with spongiform change and focal tau accumulations Q227X - numerous unicentric and multicentric PrP amyloid plaques and neurofibrillary tangles, no PrP-CAA or spongiosis

Surveys of the Broad Institute's Genome association database (gnomAD v3.1.2) and UK Biobank (Genebass.org) revealed other truncation mutations (and frameshift (fs) mutations resulting in premature termination) of *PRNP* for which there were no pathogenic reports. These were typically in the N-terminal half of PrP, including at residues 37, 41, 69 (two fs x14), 75, 81, and 131. Truncation mutations further along towards the C-terminal not obviously associated with pathogenesis were also reported at residues 186, 226 and 229. Q229X was reported in a female under 30 years of age. Q186X has been reported to be pathogenic, but no clinical data is available.^20^ In UK Biobank samples, truncation and frameshift mutations were seen at codons 7, 31, 35 (fs), 37 (x5), 49, 73 (x2), 75 (fs x2), 83 (fs x2), 91, 124 (fs x3), 128 and 196 (x3). These data are consistent with truncation becoming pathogenic when 3’ to codons 131–144. C-terminal truncations which abolish the GPI anchor and yet leave sufficient PrP to form stable beta sheet folds mediate gain of function, whereas N-terminal truncating variants simply cause a hemizygous loss of PrP function which is not associated with any known phenotype. It is interesting, however, that three p.Glu196Ter mutations were documented out of 268,749 individuals (1 in 89,583) in UK Biobank and yet this has not been recorded by prion disease specialist clinics in the UK. Several explanations of this pattern are possible, including partial penetrance, a complex relationship between truncation codon and pathogenicity, and poor ascertainment of this unusual disease.

Gain of function in C-terminal truncation mutations may be mediated by mislocalization following loss of the GPI-anchor – it has been proposed that anchorless PrP is able to spread through interstitial fluids and vascular compartments leading to peripheral and parenchymal deposition of PrP amyloid. This has been demonstrated in cell culture models and transgenic mouse models lacking the GPI anchor, where it has been observed that anchorless PrP does not localize to the cell membrane,^21^ and PrP-scrapie amyloid accumulates in both brain and peripheral organs following scrapie infection, with lack of clinical signs and spongiosis.^22,23^

Truncation of the C-terminal domain may also mediate gain of function by altering propensity of the protein to aggregate. The N-terminal domain of PrP is an intrinsically disordered region, and intrinsically disordered proteins are able to undergo liquid-liquid phase separation, which can become aberrant in neurodegenerative disease.^24^ In vitro the prion protein variant Y145X spontaneously phase-separates into liquid droplets under physiological conditions, and these liquid-like condensates gradually mature into solid-like self-replicable amyloids. Aberrant phase-transition such as this was not demonstrated in the full-length protein.^25^

Studying truncation mutations of prion protein may also allow further understanding of mechanisms of routes of spread of amyloid aggregates from the periphery to the CNS. Mice peripherally seeded with amyloid-β tend to develop mostly CAA rather than the parenchymal amyloid-β deposits that are seen in these mice as they age,^26^ which has interesting correlates with the PrP-CAA noted in cases of truncation mutations.

### Conclusion

The clinicopathological features detailed in this pedigree strengthens the evidence of an association between *PRNP* truncation mutations and the PrP systemic amyloidosis phenotype. Given that this phenotype is so different from the fCJD, GSS, and FFI phenotypes typically associated with IPD, it may be that other patients with truncation mutations of PrP are under-diagnosed. This is especially the case as many affected individuals can have symptoms attributed to peripheral PrP amyloidosis for extended periods of time before the onset of cognitive decline which usually prompts the clinician to consider prion disease as a possibility. As such, *PRNP* sequencing should be considered in cases of familial peripheral dysautonomia and neuropathies that elude diagnosis.^27^ Further study and characterization of truncation mutations in prion proteins has importance in wider understanding of the pathogenesis of neurodegenerative disease.
